# Central expression of synaptophysin and synaptoporin in nociceptive afferent subtypes in the dorsal horn

**DOI:** 10.1038/s41598-019-40967-y

**Published:** 2019-03-12

**Authors:** Jumi Chung, John F. Franklin, Hyun Joon Lee

**Affiliations:** 1 Department of Neurobiology and Anatomical Sciences, University of Mississippi Medical Center, Jackson, MS 39216 USA; 2 Research Service, G.V. (Sonny) Montgomery VA Medical Center, Jackson, MS 39216 USA; 3 School of Medicine, University of Mississippi Medical Center, Jackson, MS 39216 USA

## Abstract

Central sprouting of nociceptive afferents in response to neural injury enhances excitability of nociceptive pathways in the central nervous system, often causing pain. A reliable quantification of central projections of afferent subtypes and their synaptic terminations is essential for understanding neural plasticity in any pathological condition. We previously characterized central projections of cutaneous nociceptive A and C fibers, selectively labeled with cholera toxin subunit B (CTB) and Isolectin B4 (IB4) respectively, and found that they expressed a general synaptic molecule, synaptophysin, largely depending on afferent subtypes (A vs. C fibers) across thoracic dorsal horns. The current studies extended the central termination profiles of nociceptive afferents with synaptoporin, an isoform of synaptophysin, known to be preferentially expressed in C fibers in lumbar dorsal root ganglions. Our findings demonstrated that synaptophysin was predominantly expressed in both peptidergic and IB4-binding C fiber populations in superficial laminae of the thoracic dorsal horn. Cutaneous IB4-labeled C fibers showed comparable expression levels of both isoforms, while cutaneous CTB-labeled A fibers exclusively expressed synaptophysin. These data suggest that central expression of synaptophysin consistently represents synaptic terminations of projecting afferents, at least in part, including nociceptive A-delta and C fibers in the dorsal horn.

## Introduction

Nociceptive afferents are groups of dorsal root ganglion (DRG) neurons that deliver peripheral sensory inputs to central nociceptive pathways. Plasticity of nociceptive afferents can occur in response to injury at any part of the nervous system and increases excitability of central signal processing i.e. central sensitization, often resulting in pain^1,2^. Sprouting of primary afferents is a mechanism of plasticity that generates new branches of centrally projecting axons, and potentially their synaptic terminals, in the dorsal horn, contributing to central sensitization.

Central projections of nociceptive afferents have been investigated in the dorsal horn using immunohistochemistry for afferent subtype specific antigens, e.g. calcitonin gene-related peptide (CGRP) and tropomyosin receptor kinase A (TrkA), or using isolectin B4 (IB4) binding assay^3–7^. Transganglionic axon tracers have been injected to nerve branches to label nerve-specific afferent subtypes, e.g. cholera toxin subunit B (CTB) for myelinated A fibers and IB4 for unmyelinated C fibers^8–10^. These studies vigorously demonstrated topographical distributions of central afferent projections and quantified them, as immunoreactive areas or dorsal horn areas that encompass all projections, in healthy and in diseased states. However, much less is known about the extent to which sprouting nociceptive afferents undergo synaptogenesis, which may be necessary to develop hyperexcitability of central pain signal processing.

Synaptophysin, also known as protein p38, is a major synaptic protein that constitutes small vesicle membranes and presents in virtually all presynaptic endings^11^. An isoform, synaptoporin, with 58% homologous amino acid sequence, has been identified in small synaptic vesicles^12^. Expression patterns of synaptophysin and synaptoporin have been extensively investigated in brain subregions^13,14^ and in lumbar DRGs^15^. As these reports have collectively demonstrated, synaptophysin is more widely distributed in most areas of the nervous system, whereas synaptoporin expression was restricted in some subareas e.g. forebrain^14^ and lamina I-II of the spinal cord^15^. In DRGs at L4-L5 spinal levels, synaptophysin was expressed in both small and large diameter neurons, whereas synaptoporin was expressed almost exclusively in small diameter neurons^15^. These authors showed that a larger number of small diameter neurons (approximately 68%) express synaptoporin than those neurons (approximately 45%) that express synaptophysin, suggesting synaptoporin as a better synaptic marker for small diameter DRG neurons, supposed to be unmyelinated C fibers. However, central expression of those synaptophysin isoforms in projecting DRG neurons has not been quantitatively analyzed in the dorsal horn.

We have previously used synaptophysin to estimate synaptic terminations of cutaneous afferents of a multi-segmental nociceptive reflex, the cutaneus trunci muscle (CTM) reflex^9^. Electrical stimulations to each dorsal cutaneous nerve (DCN) from cervical (C4) to lumbosacral (S1) spinal segments produced CTM reflex responses that comprise an (A fiber mediated) early and a (C fiber mediated) late response^9,16,17^. Axon tracers, CTB and IB4, have been injected into thoracic (T7 and T13) DCNs to selectively label myelinated A fibers and unmyelinated C fibers respectively. We have shown that different central projections (i.e. immunoreactive areas) of labeled DCN afferents contributed to segmental differences of evoked CTM reflex responses depending on which DCN (T7 vs. T13) was stimulated. Synaptophysin double-labeling showed that the numbers of synaptophysin-positive terminals of DCN afferent subtypes were proportional to the immunoreactive areas of each afferent subtype across both spinal levels^9^. This suggests that central projection areas of nociceptive afferents reflect the extent of their synaptophysin-positive synaptic terminations, which may ultimately determine the size of physiological inputs to central pain signal processing. Based on the previous report that synaptoporin predominated in C fibers of the DRGs^15^, however, further investigations of the central expression of synaptoporin in those DCN afferents are required.

In the current studies, distribution patterns of synaptoporin and synaptophysin have been characterized in the thoracic (T7 and T13) spinal cord and compared in superficial laminae of the dorsal horn, where cutaneous nociceptive C fibers project. Using double-labeling quantification, we analyzed overlapping areas between synaptic markers (synaptophysin or synaptoporin) and C fibers that include peptidergic (CGRP positive) and non-peptidergic (IB4-binding) subpopulations. Finally, expressions of synaptoporin in DCN-specific afferent projections labeled with CTB and IB4 injections were compared to the expression patterns of synaptophysin in those cutaneous-specific afferents shown in our previous report^9^. The goals of current studies are answering following questions: (1) whether synaptoporin is a dominant synaptic molecule of C fibers in the thoracic dorsal horn as seen in the lumbar DRGs^15^, (2) whether the central expression of synaptophysin isoforms depends on nociceptive afferent subtypes such as A fibers, peptidergic C fibers, IB4 binding C fibers, and (3) what isoform of the synaptophysin family labels most synaptic terminals of cutaneous nociceptive afferent subtypes. Although a single synaptic vesicle molecule cannot represent all synaptic vesicles across neuronal populations, the use of a general synaptic marker enables an estimation of putative synaptic termination of projecting afferents with reasonable efforts, especially for time-consuming immunohistochemical analysis.

## Results

### Distribution of synaptophysin and synaptoporin in thoracic dorsal horns

**Figure 1:**
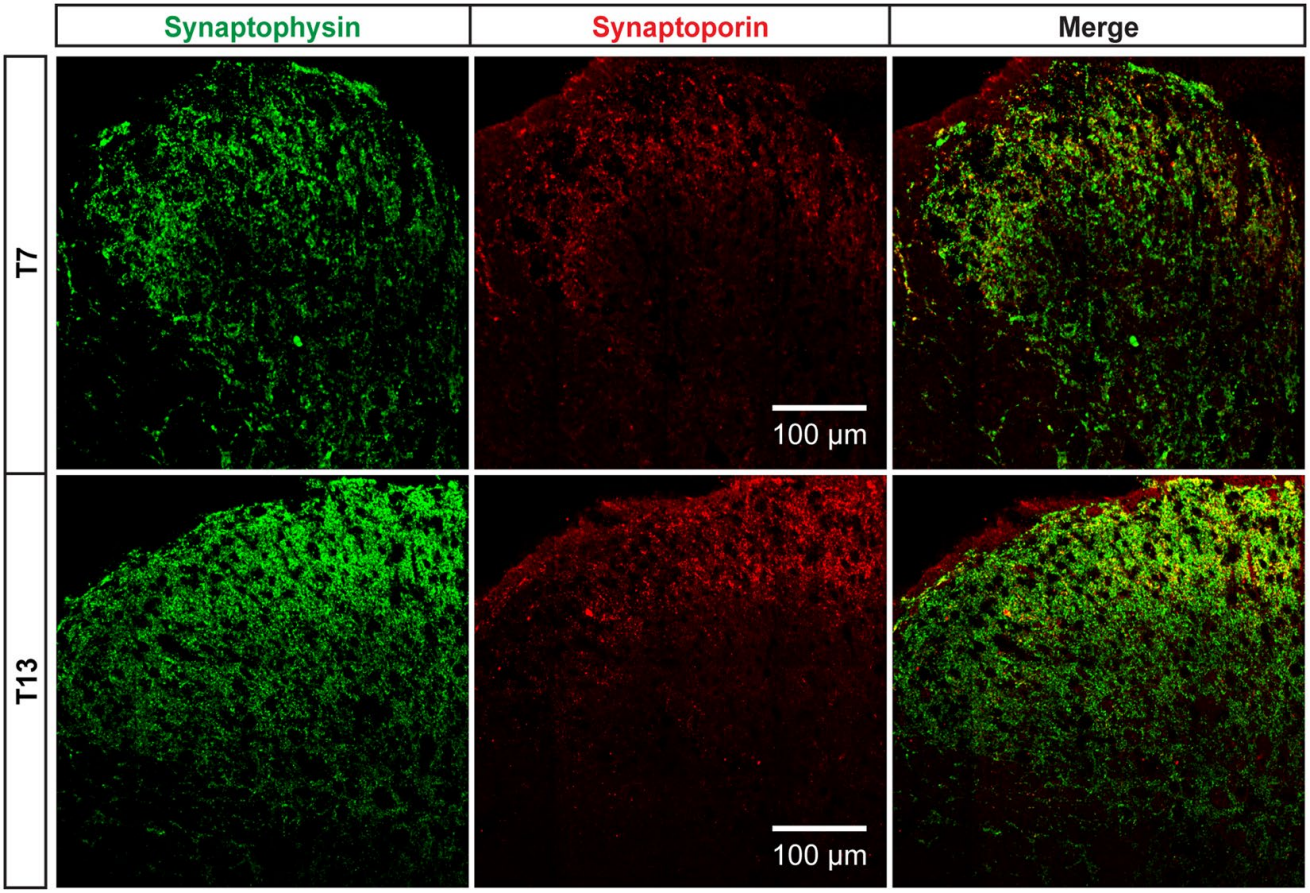
Central distributions of synaptophysin and synaptoporin in the T7 and T13 spinal cord dorsal horn in rats. Immunohistochemistry for presynaptic vesicle proteins, synaptophysin (green) and synaptoporin (red), shows that synaptophysin is distributed across dorsal horn laminae whereas synaptoporin is localized in superficial laminae I-II. Double-labeling of synaptic terminals positive for both synaptophysin and synaptoporin is visualized as yellow in merged images. Shown are collages of 16 confocal images at 40X objective lens.

We first developed the laminar distribution profiles of synaptophysin and synaptoporin in the dorsal horn of T7 and T13 spinal cord, where we have characterized the central projection patterns of DCN C fibers^9^. Consistent with previous reports using *in-situ* hybridization^14^ and immunohistochemistry^15^, synaptoporin was exclusively distributed in superficial laminae I-II, whereas synaptophysin showed a broad distribution in both T7 and T13 dorsal horns (Fig. 1). Merged representative images demonstrated that the broad laminar distribution of synaptophysin covered up the superficial laminae where synaptoporin was exclusively expressed. There was double-labeling (yellow dots in Fig. 1) between these two synaptic vesicle molecules in that overlaid area.

**Figure 2:**
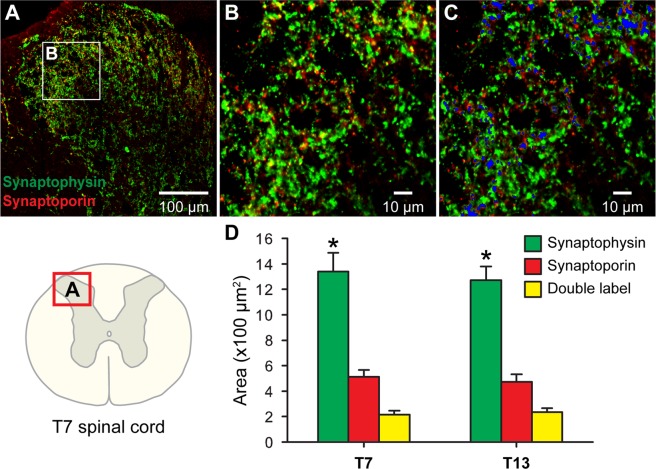
Quantitative analysis of synaptophysin, synaptoporin, and their co-localization in the T7 and T13 spinal cord dorsal horn. A representative montage image (**A**) was composed of 16 confocal images from the dorsal horn as marked in a T7 spinal cord diagram (lower left corner). A confocal image was chosen in lateral superficial laminae I-II as a region of interest (white box inset in **A**,**B**). The co-localization of synaptophysin (green) and synaptoporin (red) was shown in yellow (**B**) and overlaid with blue masks (**C**) of which areas were measured for double labels. Immunoreactive areas of synaptophysin, synaptoporin, and the double labels were quantified in T7 and T13 dorsal horns. (**D**) Asterisks indicate significant differences between synaptoporin and synaptophysin at each thoracic level (n = 4, *p* < 0.05, Wilcoxon rank sum test, Cohen’s d > 1.3).

Immunoreactive areas for synaptophysin, synaptoporin, and their double-labeling were quantified in the region of interest at the lateral border of T7 and T13 dorsal horns (Fig. 2A) where DCN-specific C fibers project^9^. Immunoreactive areas of synaptophysin were significantly larger than synaptoporin-positive areas at both T7 and T13 spinal levels (Fig. 2D). Double-labeled areas overlapped much greater areas positive for synaptoporin alone (44.24% ± 8.56 (standard deviation, SD) at T7 and 46.76% ± 7.96 at T13) than areas positive for synaptophysin alone (15.63% ± 2.27 at T7 and 16.41% ± 3.63 at T13). These data demonstrated that synaptophysin was a dominant presynaptic molecule in laminae I-II of thoracic dorsal horns, where nociceptive C fibers terminate.

### Expression of synaptophysin and synaptoporin in peptidergic C fibers

**Figure 3:**
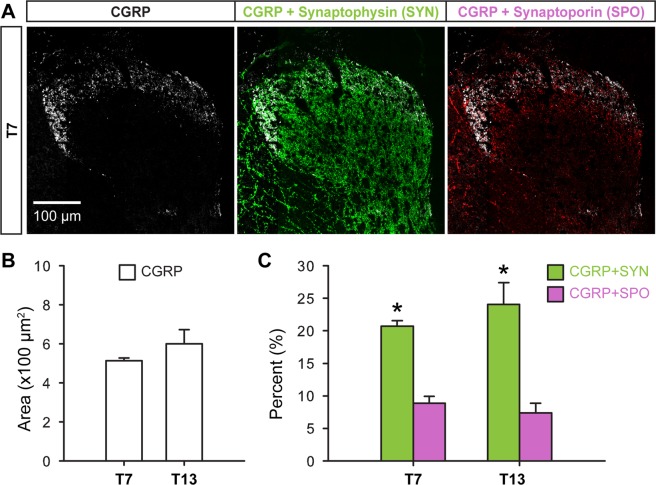
Co-localization of synaptophysin and synaptoporin with peptidergic C fibers (CGRP) in T7 and T13 dorsal horns. A representative montage image shows CGRP+ C fibers distributed in laminae I-II in the T7 dorsal horn. (**A**) Those peptidergic C fibers were quantified in T7 and T13 dorsal horn. (**B**) Double labels of CGRP with synaptophysin or with synaptoporin were quantified and normalized to the CGRP positive areas. (**C**) There were significant differences between CGRP areas co-labeled with synaptophysin and those CGRP areas co-labeled with synaptoporin both in T7 and T13 (n = 4, *p* < 0.05, Kruskal-Wallis test with multiple comparison test, Cohen’s d > 1.3).

We next investigated which synaptic vesicle molecule, synaptophysin or synaptoporin, is expressed to a greater extent in nociceptive C fiber subtypes that project in thoracic dorsal horns. To label synaptic terminals of peptidergic C fibers, spinal cord tissues were subjected to triple immunohistochemistry for CGRP, synaptophysin, and synaptoporin (Fig. 3A). Consistent with the typical distribution of CGRP in rats^3,9,18^, peptidergic C fibers were found mainly in the superficial dorsal horn from lamina I to the outer layer of lamina II at both T7 and T13. There was double-labeling of CGRP with both synaptophysin or synaptoporin. Quantitative analysis in the region of interest (see Fig. 2A) produced immunoreactive areas of CGRP+ C fibers (Fig. 3B) and their percent colabeling with synaptophysin and synaptoporin (Fig. 3C). Significantly greater areas of CGRP+ C fibers were co-labeled with synaptophysin than with synaptoporin at T7 and T13.

### Expression of synaptophysin and synaptoporin in IB4-binding C fibers

**Figure 4:**
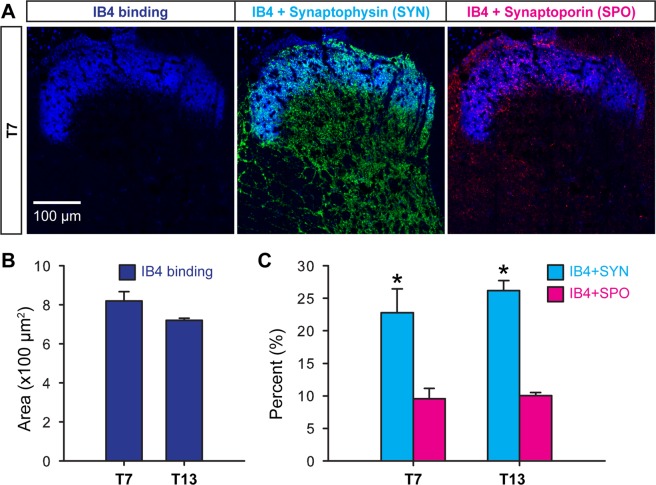
Co-localization of synaptophysin and synaptoporin with IB4-binding C fibers in the thoracic dorsal horn. IB4 binding C fibers distributed in lamina II of T7 and T13 dorsal horns and expressed synaptophysin and/or synaptoporin as shown in a representative montage of confocal images from T7. (**A**) Areas of IB4 binding projections within the investigated region of interest were analyzed at both thoracic levels.(**B**) Colocalization of IB4 binding axon terminals with synaptophysin or with synaptoporin was assessed by double labeling quantification (**C**) to find significantly different expression levels of synaptoporin and synaptophysin in this C fiber subpopulation (n = 4, *p* < 0.05, Kruskal-Wallis test with multiple comparison test, Cohen’s d > 1.3).

Another population that includes approximately 50% unmyelinated DRG neurons is non-peptidergic and binds to IB4^19,20^. To label those non-peptidergic C fibers, spinal cord sections adjacent to the sections used for CGRP immunohistochemistry were subjected to the IB4-binding assay, followed by triple immunohistochemistry for IB4, synaptophysin, and synaptoporin (Fig. 4A). A thick band of IB4 labeling was found in the superficial laminae at both T7 and T13, where IB4-binding C fibers were supposed to project^7,19,20^.

Immunoreactive areas of IB4-binding C fibers were quantified in the region of interest at the lateral dorsal horns (Fig. 4B). Interestingly, IB4-binding areas tended to be larger than CGRP+ areas (Fig. 3B) at both T7 and T13, suggesting that IB4-binding assay labels non-peptidergic C fibers to a greater extent than immunohistochemistry for CGRP does peptidergic C fibers. Colabeling ratios of IB4-binding C fibers with synaptophysin was significantly higher than those ratios with synaptoporin (Fig. 4C). Collectively, these data demonstrated that both C fiber populations, peptidergic and IB4-binding C fibers, predominantly expressed synaptophysin at the dorsal horn of the thoracic spinal cord.

### Expression of synaptophysin and synaptoporin in DCN-specific IB4+ C fibers

**Figure 5:**
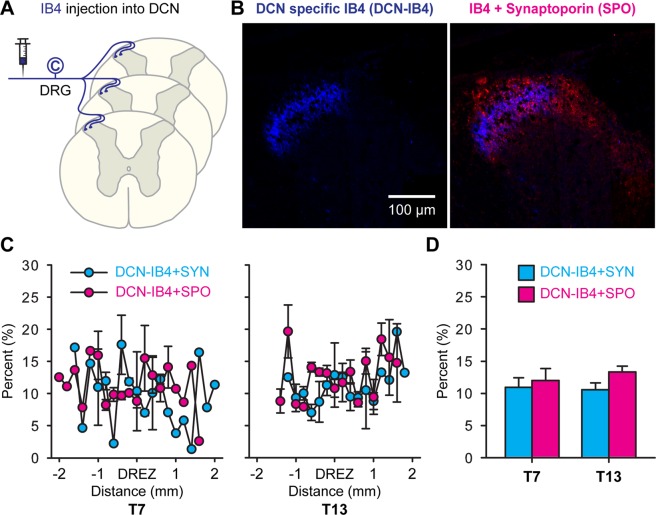
Expression of synaptophysin and synaptoporin in dorsal cutaneous C fibers in the T7 and T13 spinal cord. Cutaneous C fibers were labeled with IB4 injections into DCNs. (**A**) DCN-specific C fibers projected laterally at the inner layer of laminae II overlaid by the synaptoporin distribution area as shown in representative montages of confocal images at T7. (**B**) Overlapping areas of DCN-specific IB4+ C fibers with synaptoporin and synaptophysin were quantified at every 200 μm across the sections centered at the dorsal horn entry zone (DREZ) at T7 and T13. (**C**) Bar graphs show averaged percent overlapping areas across those serial sections from 3 rats (**D**).

We have previously estimated putative synaptic termination of DCN-specific IB4+ C fibers using synaptophysin at both T7 and T13^9^. To investigate the expression of synaptoporin in those cutaneous-specific IB4+ C fibers, adjacent serial spinal cord sections inherited from the previous studies in rats with peripheral injections of IB4 (Fig. 5A) were used in the current study. DCN-specific IB4+ C fibers were labeled in apparently smaller areas, limited to the lateral corner of lamina II (Fig. 5B), when compared to C fibers labeled by IB4-binding assay (Fig. 4A), shown in those representative images at T7. Merged images clearly demonstrated that DCN-specific IB4+ C fibers projected exclusively in the mid-laminar (lamina II) part of synaptoporin+ areas (Fig. 5B) which, in contrast, encompassed most laminar areas of IB4-binding C fibers (Fig. 4A).

Double-labeled immunoreactive areas were measured in the region of interest of serial sections at an interval of 200 µm, centered at dorsal root entry zone (DREZ), to calculate percent terminations of DCN-specific IB4+ C fibers (Fig. 5C). The areas of double-labeling were then averaged across those sections at T7 and T13 (Fig. 5D). Percent colocalizations of DCN-specific IB4+ C fibers with synaptophysin or synaptoporin were comparable with each other at both T7 and T13 (Fig. 5), in contrast to the significantly different colabeling ratios for CGRP+ (Fig. 3C) and IB4-binding C fibers (Fig. 4C) with those synaptic vesicle molecules being greater with synaptophysin. These data demonstrated that DCN-specific IB4+ C fibers showed distinct expression patterns of synaptic vesicle molecules from the expression patterns in non-DCN IB4-binding C fibers in the dorsal horn.

### Synaptoporin expression in DCN-specific A fibers

**Figure 6:**
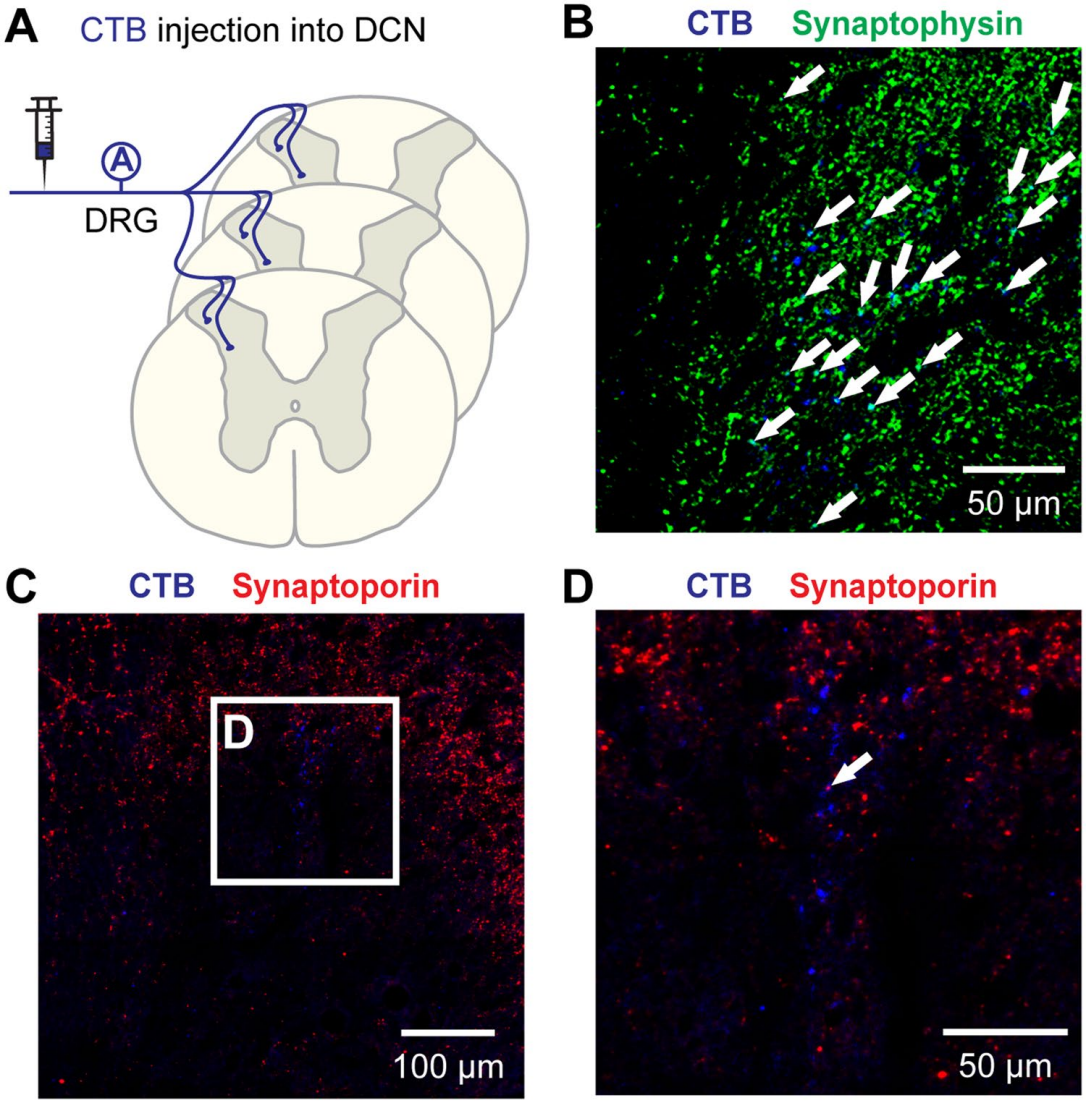
Co-localization of synaptophysin and synaptoporin with cutaneous A fibers in the thoracic spinal cord dorsal horn. Central projections of cutaneous A fibers were labeled with CTB injections into DCNs. (**A**) DCN-specific CTB+ A fibers projected in laminae III-V (**B**) where many of them were co-localized with synaptophysin (arrows) as shown in our previous report. Some projections of CTB+ A fibers were found in the area where synaptoporin was expressed (**C**) but only few co-localizations were found (arrow in **D**). Panel C is a montage of 16 confocal images.

In the previous report^9^, synaptophysin has been used to estimate putative terminations of A fibers specifically labeled by CTB injections into T7 and T13 DCNs (Fig. 6A). Most CTB+ A fibers were distributed in deeper laminae III-V and a large number of those immunoreactive particles expressed synaptophysin (arrows in Fig. 6B). In addition to our previous report on the number of CTB+ particles (approximately 40%) that co-expressed synaptophysin at T7 and T13^9^, immunoreactive areas double-labeled with CTB and synaptophysin were measured in the current study, showing percent colabeling of 18.64% ± 7.30 (SD) at T7 and 16.22% ± 5.38 at T13. However, only a few CTB+ A fibers were found in laminar areas where synaptoporin was concentrated (Fig. 6C), and colabeling with synaptoporin was rarely found on some serial sections if any (arrows in Fig. 6D). Quantification for double labeling was not performed. Taken all together, synaptophysin was a better representative synaptic marker for DCN-specific CTB+ A fibers and IB4+ C fibers than synaptoporin at T7 and T13 dorsal horns.

## Discussion

In this study, we have established the expression profiles of two synaptic vesicle protein isoforms, synaptophysin and synaptoporin, in T7 and T13 spinal cord dorsal horns, comparing their colocalization with central projections of nociceptive afferent subtypes. We have pursued this comparison to investigate what nociceptive afferent subtypes express which synaptic vesicle molecules and to find the most representative synaptic molecules in the central projections of those afferent subtypes. Part of the motivation to establish the central expression profiles of general synaptic molecules was to understand the complex biology of nociceptive DRG neurons and their central terminations in order to find any changes of afferent terminations in animal models with pathophysiological conditions of pain (e.g. following neural injury) in future studies.

Nociceptive afferents include thinly myelinated Aδ fibers and unmyelinated C fibers. Nociceptive C fibers are divided into two sub-classes: peptidergic and non-peptidergic C fiber populations. Those DRG neurons with unmyelinated small diameter axons express TrkA, a receptor for nerve growth factor (NGF), and neuropeptides such as CGRP and substance P during development^21,22^. Approximately one half of those C fibers in the DRG express Ret, another tyrosine kinase receptor that binds glial cell-derived neurotrophic factor (GDNF), replacing TrkA at a late embryonic or early postnatal stage^23^. These cells that lose the expression of TrkA and neuropeptides change their histochemical properties, binding to IB4^20,23^. To include all C fiber projections, we therefore performed CGRP immunohistochemistry and IB4-binding assay on neighboring spinal cord sections. Despite the roughly equal numbers of peptidergic or IB4-binding small diameter cell bodies in the DRG^23^, we found that IB4-binding areas (Fig. 4B) tends to be larger than CGRP+ areas (Fig. 3B) in the superficial laminae at both T7 and T13. This, in part, can be explained by our previous finding that approximately 10% DCN-specific IB4+ C fibers expressed CGRP in thoracic DRGs and dorsal horns^9^. Another possible explanation is that IB4-binding C fibers may project to a greater dorsal horn area than CGRP-bearing C fibers, as we discuss differential arborization of primary afferents below.

Over decades, significant progress has been made to discover synaptic molecules that regulate neurotransmitter metabolism, synaptic vesicle formation, axonal trafficking, and neurotransmitter recycling^24^. On the one hand, researchers have sought molecules expressed in specific subtypes of DRG neurons and have developed their distribution patterns in the dorsal horn. The best example would be the family of vesicular glutamate transporters (vGLUTs). On the other hand, a few molecules, such as synaptophysin and synapsin^11^, have been sought as general presynaptic markers.

As nociceptive afferents are mainly glutamatergic, vGLUTs have been extensively studied as specific presynaptic molecules of nociceptive DRG neurons^25^. To date, three different types of vGLUTs (vGLUT1, vGLUT2, vGLUT3) have been reported, and their expression patterns have been used to characterize glutamatergic neuron subtypes in the DRG and spinal cord^25–29^. Based on the laminar distribution profiles of vGLUTs in the dorsal horn, the expression of vGLUTs is largely dependent on afferent subtypes: CTB-labeled A fibers in laminae III-VI express vGLUT1^9,27^, IB4-binding and CGRP-bearing C fibers in laminae I-II express vGLUT2^9,27,29^, and C-low threshold mechanoreceptors (C-LTMRs) in laminae I-II express vGLUT3^28^. However, the expression profiles of vGLUTs in nociceptive afferent subtypes seem to be variable at the borders of laminae and subject to change in pathological conditions. For instance, sympathetic preganglionic neurons in the intermediolateral nucleus increased expression of vGLUT1 instead of vGLUT2 after transection spinal cord injury, which was associated with the development of autonomic dysfunction after that injury^30^. Therefore, the use of vGLUTs for an estimation of synaptic termination will require multiple cross labels between vGLUTs in different afferent subtypes. This suggests a need for a general presynaptic marker that labels the most nociceptive afferent projections for the purpose of reliable quantification.

Due to the extensive expression of synaptophysin in almost all synaptic vesicles^11^, numerous studies have used synaptophysin as a general presynaptic marker across the central nervous system (CNS). We have also used synaptophysin to estimate putative synaptic termination of DCN-specific nociceptive afferents in the thoracic dorsal horn^9^. As a previous study in the lumbar DRGs demonstrated, however, synaptoporin was preferentially expressed over synaptophysin in unmyelinated, small diameter neurons, including both peptidergic (CGRP+) and non-peptidergic (IB4-binding) C fibers^15^. This suggested a hypothesis that synaptoporin may be a dominant presynaptic molecule in the central projection of unmyelinated C fiber populations in the thoracic dorsal horn. Our findings disqualified this hypothesis by demonstrating that immunoreactive areas of synaptophysin predominated in a wider area of the thoracic dorsal horn (Fig. 1) especially in the region of interest of laminae I-II (Fig. 2) where nociceptive C fibers project and that projections of both CGRP-bearing (Fig. 3) and IB4-binding (Fig. 4) afferents preferentially expressed synaptophysin in those superficial laminae I-II.

The different expression of synaptic molecules between lumbar DRGs and thoracic dorsal horns may be simply due to segmental variation (lumbar vs. thoracic spinal levels). Aside from varying expression levels during development, the widespread laminar distribution of synaptophysin was homogeneous throughout the adult dorsal horn, as consistently seen in the cervical^31^, thoracic (Fig. 1), and lumbar^15,31^ spinal cord. The exclusive distribution of synaptoporin in laminae I-II was also consistent at the thoracic (Fig. 1) and lumbar^15^ spinal cord. In the current studies, we quantitatively compared central expression profiles of synaptophysin isoforms at T7 and T13, which were 6 spinal segments away from each other. Synaptophysin and synaptoporin showed homogeneous profiles in terms of immunoreactive areas of each isoform and their colabeling ratios when compared with both thoracic levels (Fig. 2D). Their expression ratios in different C fiber (CGRP-bearing and IB4-binding) populations were also steady at those levels (Figs 3, 4). Collectively, these suggest that synaptophysin isoforms are less likely to reverse their central expression profiles, at least at the lateral superficial laminae in the lumbar dorsal horn, in contrast to the thoracic dorsal horns. This idea that synaptophysin may predominate in C fiber projections in the lumbar dorsal horn remains a conflict with the fact that those C fibers preferentially express synaptoporin in lumbar DRGs^15^.

Centrally projecting nociceptive afferents branch at the DREZ, travel along the rostral and caudal axis, and arborize within specific dorsal horn laminar areas corresponding to afferent subtypes. Intra-axonal injections of labels into electrophysiologically identified individual afferent axons have been extensively used to determine their topographical representation in the dorsal horn^32,33^. These studies demonstrated, however, that individual afferents, even in the same physiological property groups, had a wide variety of arborization patterns in terms of rostral/caudal, lateral/medial, and laminar distribution. In addition, unmyelinated C fibers and thinly myelinated Aδ fibers form two different types of synaptic glomeruli, where their individual axon can be involved in a few synapses, being presynaptic to dendritic spines or postsynaptic to axoaxonic synapses^34–36^. These various ultrastructural synaptic arrangements may differentiate numbers of synaptic terminals of each arborizing axon. Due to the heterogenous arborization pattern and the formation of complex synaptic glomeruli, there will be no simple linear relationship between the number of DRG neurons and the extent of their central termination. This supports the possibility that the expression patterns of synaptophysin isoforms in DRG neurons could reverse their central expression in the dorsal horn at any spinal (lumbar and/or thoracic) levels.

A novel finding in the current studies was that the expression profiles of synaptophysin isoforms in DCN-specific C fibers differed from those of generic C fibers. Compared to IB4-binding C fibers (Fig. 4), colabeling of DCN-specific IB4+ C fibers with synaptophysin remarkably decreased to the level of those co-labeled with synaptoporin (Fig. 5). IB4 specifically binds terminal α-D-galactosyl residues of glycoproteins^37^ and IB4-binding labels not only non-peptidergic C fibers, but also blood vessel endothelial cells^38^ and microglia^39^ on nervous tissues. This means that the IB4-binding areas measured in the dorsal horn included non-neuronal labeling (Fig. 4B). Because any of the synaptophysin isoforms is unlikely expressed in endothelial cells and microglia, a removal of potential non-neuronal binding from the total IB4-binding areas (denominator) may slightly increase the percent colabeling ratios for both isoforms (Fig. 4C). In contrast, peripheral injections of IB4 into DCNs of selectively labeled C fibers produce no such non-neuronal labeling (Fig. 5). Thus, despite the different approach of using IB4-binding assay or IB4 injection, our findings demonstrated that cutaneous-specific IB4-binding C fibers expressed both synaptophysin isoforms to a roughly equal extent. Due to the lack of appropriate transganglionic axon tracer for peptidergic C fiber, we could not analyze the central projection of DCN-specific peptidergic C fibers in the dorsal horn. However, the preferential expression of synaptophysin was generally shown in C fibers, including both peptidergic (Fig. 3C) and IB4-binding (Fig. 4C) C fiber subpopulations. Taken all together, the expression profiles of synaptophysin isoforms in centrally projecting nociceptive afferents is dependent on the origin of afferents (e.g. cutaneous vs. non-cutaneous C fibers) rather than the afferent subtype (e.g. peptidergic vs. IB4-binding C fibers), at least, for IB4-binding cutaneous C fibers.

Due to the lack of data (e.g. numbers of synaptophysin+/synaptoporin+ neurons in thoracic DRGs and central projection profiles of synaptophysin isoforms in the lumbar dorsal horn), we could not clearly demonstrate whether the expression levels of synaptic molecules in the DRG reflect their central expression in the dorsal horn. Despite the ongoing loose relationship between the nociceptive DRG neurons and their central projections, and between the complex arborization and synaptic termination, profiling the central expression of synaptic molecules in the CNS will be much more informative than their expressions in DRGs. It is in our best interest to relate them to the final physiological outcomes mediated by those nociceptive afferents. Thus, a reliable and quantifiable synaptic molecule that colocalizes in most terminals of nociceptive afferent subpopulations will be required for the precise assessment of central terminations, with a reasonable time and effort, to understand the central mechanisms of pain signal processing in health and disease. Our data suggest that synaptophysin is a reliable synaptic marker for nociceptive C fiber populations when double labeled with C fiber-specific antigens in the superficial dorsal horn. They also recommend the use of synaptophysin for primary afferent subpopulations selectively labeled with the combination of nerve-specific transganglionic tracers, IB4 and CTB, which enables the comparison between A and C fibers.

## Methods

All animal procedures were performed at our former institution, Emory University, and the remaining work on tissue sections harvested from the animals was completed at the University Mississippi Medical Center.

### Animals

Animal procedures were reviewed and approved by the Institutional Animal Care and Use Committee at Emory University. All experiments were performed in accordance with relevant guidelines and regulations. Female Long Evans rats (200–225 g) were purchased from Charles River Laboratories International, Inc. (Wilmington, MA) and pair-housed in standard transparent plexiglas cages. Two groups of Long Evans rats were used, one group (n = 3) for transganglionic tracer injections with IB4 and CTB and the other group (n = 4) without tracer injections. Animals were kept in a regular 12-hour light-dark cycle and *ad libitum* diet was provided.

### Transganglionic tracer injection

DCN labeling using transganglionic axonal tracers was performed as described in our previous report^9^. Briefly, rats (n = 3) were anesthetized with intraperitoneal injections of Ketamine (75 mg/kg, Bioniche, Morgantown, WV)/Dexmedetomidine (0.25 mg/kg, Dexdomitor, Pfizer, New York, NY) and DCNs at T7 and T13 spinal levels were exposed through a skin incision along the median line across thoracic areas. Transganglionic axonal tracers, IB4 (Vector Laboratories, Burlingame, CA) and CTB (List Biological Laboratories, Campbell, CA) were dissolved in sterile phosphate buffered saline (PBS; pH 7.4) for injections (1 ul of 2% solution for each tracer). IB4 was injected into left DCNs and CTB into right DCNs, at both T7 and T13 spinal levels. A 30-gauge needle with a Hamilton syringe (75RN, Hamilton, Reno, NV) was inserted into a proximal site of each DCN, and injections were made using a motorized pump (Model #310, Stoelting Co., Wood Dale, IL) for over 1 minute. Then, the skin was closed with surgical staples. Animals were recovered from anesthesia with a reversal drug, Antisedan (2 mg/kg, Pfizer), and they received post-operative care including saline injections as needed with daily monitoring.

### Spinal cord tissue processing

Three days after transganglionic axon tracer injections, animals were euthanized (150 mg/kg, Euthasol, Virba, Fort Worth, TX) and perfused transcardially with heparinized PBS and 4% Paraformaldehyde. The other group of age-matched animals without transganglionic tracing were also sacrificed for tissue harvesting. The spinal cord segments at T7 and T13 were harvested and cryoprotected in 30% sucrose at 4 °C for 24 hours. The cords were then embedded in OCT medium (Optimal Cutting Temperature, Tissue-Tek, Torrance, CA) and stored at −80 °C. Serial 20 μm-thick sections were cut from 1 cm-long tissues centered at T7 and T13 spinal segments with cryostat (Leica CM1900, Leica Biosystems Inc., Buffalo Grove, IL).

### Immunohistochemistry and confocal microscopy

Immunohistochemistry was performed to visualize CTB+ A and IB4+ C fibers, synaptophysin, synaptoporin, and CGRP. Briefly, sections were incubated for 1 hour in PBS with 10% normal donkey serum (NDS) and 0.3% Triton X-100 to block nonspecific reagent reactions. Sections were then incubated overnight at 4 °C in primary antibody solution diluted in PBS with 1% NDS and 0.3% Triton X-100. For the IB4-binding assay on tissues without DCN-specific IB4 labeling, additional incubation with IB4 (50 ng/μl) for 1 hour at room temperature was done before primary antibody incubation. The primary antibodies included goat anti-IB4 (1:400, Vector, Burlingame, CA), goat anti-CTB (1:2000, List Biological Laboratories, Campbell, CA), mouse anti-synaptophysin (1:1000, Synaptic Systems), rabbit anti-synaptoporin (1:300, Synaptic Systems), and Guinea pig anti-CGRP (1:2000, Peninsula Laboratories International, San Carlos, CA). On the following day, sections were rinsed and incubated with corresponding fluorescent-labeled secondary antibody for 1 hour at room temperature: donkey anti-goat IgG Cy3 (1:400, Jackson ImmunoResearch), donkey anti-mouse IgG 488 (1:400, Jackson ImmunoResearch), donkey anti-rabbit IgG 647 (1:400, Jackson ImmunoResearch), and donkey anti-guinea pig Cy3 (1:250, Jackson ImmunoResearch). After rinsing, the sections were mounted with mounting media (Vectashield, Vector Laboratories) which contained DAPI.

T7 and T13 dorsal horn images were taken at 40X magnification using a spinning disk confocal microscope system (Diskovery, BioVision Technologies, Inc., Exton, Pa) and a CCD digital camera (Hamamatsu Photonics, Hamamatsu City, Japan) connected to Metamorph software (Molecular Devices, Sunnyvale, CA). To capture a representative view of the entire dorsal horn, 16 confocal images (512 × 512 pixels) were montaged using ImageJ software (NIH, Bethesda, MD). For immunoreactive area measurement, a single confocal image (512 × 512 pixels, 13,916 μm^2^) was taken at 40X magnification as a region of interest at the lateral corner of laminae I-II where DCN-specific IB4+ C fibers project^9^.

### Immunoreactive area measurement in the region of interest

The immunoreactive areas of IB4, CTB, synaptoporin, synaptophysin, and CGRP were analyzed with ImageJ as described previously^9,40–42^. Briefly, confocal images were converted to 8-bit images and an optimal threshold grey value was determined to select specific immunohistochemical labeling across the set of images analyzed for each antigen. Note that we have used the identical lot of secondary antibodies and the consistent setting at the confocal microscope including pinhole size, laser intensity, digital gain, and exposure time. Pixel areas selected above the threshold grey value were converted to total immunoreactive areas in each image.

To quantify the double-labeled immunoreactive area, multi-channel confocal images from sections with immunohistochemistry for 2 different antigens were separated. Each separated image underwent the thresholding method as described above. Using Color Threshold function at ImageJ, both color channel images were fused, and pixels that exceeded both threshold values were determined to measure double-labeled immunoreactive areas. Percent double-labeling areas were calculated as CGRP+ synaptophysin/CGRP and CGRP+ synaptoporin/CGRP (Fig. 3), IB4+ synaptophysin/IB4 and IB4+ synaptoporin/IB4 (Fig. 4), and DCN-IB4+ synaptophysin/DCN-IB4 and DCN-IB4+ synaptoporin/DCN-IB4 (Fig. 5). Please note that our previous publication reported double-labeled particle numbers of DCN-specific afferents with synaptophysin (e.g. DCN-IB4+ synaptophysin)^9^. We chose the double-labeling area measurements in the current studies to avoid miscounting of adjacent pixel groups (see blue masks in Fig. 2C) of which selection may vary at different thresholds. Please also note that the data of DCN-IB4+ synaptophysin/DCN-IB4 (Fig. 5C) was newly generated to be compatible with other double-labeled areas in this study.

### Statistical analysis

Data in graphs are shown as mean values with error bars indicating the standard deviation or the standard error of the mean and with the number of individual animals (n) as noted in figure legends. Statistical analyses were conducted in Matlab software (The MathWorks, Inc., Natick, MA). The Jarque–Bera test was performed to determine whether data were normally distributed. If any data in a comparison were not normally distributed, non-parametric tests, the Wilcoxon rank sum test for two groups or the Kruskal-Wallis test for more than two groups, were used as alternatives for *t*-test and analysis of variance (ANOVA) respectively. Multiple pairwise comparisons were followed by the Kruskal-Wallis test to compare between groups. Statistical significance was determined with the threshold value of *p* < 0.05 and the power for group comparisons was measured with effect size (Cohen’s d).

## Data Availability

The datasets generated during and/or analyzed during the current study are available from the corresponding author on reasonable request.

## References

[1] Walters ET (2012). Nociceptors as chronic drivers of pain and hyperreflexia after spinal cord injury: an adaptive-maladaptive hyperfunctional state hypothesis. Front Physiol.

[2] Woolf CJ, Shortland P, Coggeshall RE (1992). Peripheral nerve injury triggers central sprouting of myelinated afferents. Nature.

[3] Hou S, Duale H, Rabchevsky AG (2009). Intraspinal sprouting of unmyelinated pelvic afferents after complete spinal cord injury is correlated with autonomic dysreflexia induced by visceral pain. Neuroscience.

[4] Marsh DR, Weaver LC (2004). Autonomic dysreflexia, induced by noxious or innocuous stimulation, does not depend on changes in dorsal horn substance p. J Neurotrauma.

[5] Detloff MR (2016). Delayed Exercise Is Ineffective at Reversing Aberrant Nociceptive Afferent Plasticity or Neuropathic Pain After Spinal Cord Injury in Rats. Neurorehabil Neural Repair.

[6] Kruger L, Silverman JD, Mantyh PW, Sternini C, Brecha NC (1989). Peripheral patterns of calcitonin-gene-related peptide general somatic sensory innervation: cutaneous and deep terminations. J Comp Neurol.

[7] Bennett DL, Dmietrieva N, Priestley JV, Clary D, McMahon S (1996). B. trkA, CGRP and IB4 expression in retrogradely labelled cutaneous and visceral primary sensory neurones in the rat. Neurosci Lett.

[8] Clarke JN, Anderson RL, Haberberger RV, Gibbins IL (2011). Non-peptidergic small diameter primary afferents expressing VGluT2 project to lamina I of mouse spinal dorsal horn. Molecular pain.

[9] Lee HJ, White JM, Chung J, Tansey KE (2017). Peripheral and central anatomical organization of cutaneous afferent subtypes in a rat nociceptive intersegmental spinal reflex. J Comp Neurol.

[10] Shehab SA, Hughes DI (2011). Simultaneous identification of unmyelinated and myelinated primary somatic afferents by co-injection of isolectin B4 and Cholera toxin subunit B into the sciatic nerve of the rat. J Neurosci Methods.

[11] De Camilli P (1988). The synaptic vesicle proteins synapsin I and synaptophysin (protein P38) are concentrated both in efferent and afferent nerve endings of the skeletal muscle. J Neurosci.

[12] Knaus P, Marqueze-Pouey B, Scherer H, Betz H (1990). Synaptoporin, a novel putative channel protein of synaptic vesicles. Neuron.

[13] Fykse EM (1993). Relative properties and localizations of synaptic vesicle protein isoforms: the case of the synaptophysins. J Neurosci.

[14] Marqueze-Pouey B, Wisden W, Malosio ML, Betz H (1991). Differential expression of synaptophysin and synaptoporin mRNAs in the postnatal rat central nervous system. J Neurosci.

[15] Sun T (2006). Differential expression of synaptoporin and synaptophysin in primary sensory neurons and up-regulation of synaptoporin after peripheral nerve injury. Neuroscience.

[16] Petruska JC (2014). Organization of sensory input to the nociceptive-specific cutaneous trunk muscle reflex in rat, an effective experimental system for examining nociception and plasticity. J Comp Neurol.

[17] Theriault E, Diamond J (1988). Nociceptive cutaneous stimuli evoke localized contractions in a skeletal muscle. J. Neurophysiol..

[18] Gibson SJ (1984). Calcitonin gene-related peptide immunoreactivity in the spinal cord of man and of eight other species. J Neurosci.

[19] Kitchener PD, Lapiz MD, Wilson P, Snow PJ (1994). Transganglionic labelling of primary sensory afferents in the rat lumbar spinal cord: comparison between wheatgerm agglutinin and the I-B4 isolectin from Bandeiraea simplicifolia. Journal of neurocytology.

[20] Bennett DL, Averill S, Clary DO, Priestley JV, McMahon SB (1996). Postnatal changes in the expression of the trkA high-affinity NGF receptor in primary sensory neurons. The European journal of neuroscience.

[21] Snider WD, McMahon SB (1998). Tackling pain at the source: new ideas about nociceptors. Neuron.

[22] Fitzgerald M (2005). The development of nociceptive circuits. Nat. Rev. Neurosci..

[23] Molliver DC (1997). IB4-binding DRG neurons switch from NGF to GDNF dependence in early postnatal life. Neuron.

[24] Santos MS, Li H, Voglmaier SM (2009). Synaptic vesicle protein trafficking at the glutamate synapse. Neuroscience.

[25] Brumovsky PR (2013). VGLUTs in Peripheral Neurons and the Spinal Cord: Time for a Review. ISRN Neurol.

[26] Oliveira AL (2003). Cellular localization of three vesicular glutamate transporter mRNAs and proteins in rat spinal cord and dorsal root ganglia. Synapse.

[27] Todd AJ (2003). The expression of vesicular glutamate transporters VGLUT1 and VGLUT2 in neurochemically defined axonal populations in the rat spinal cord with emphasis on the dorsal horn. The European journal of neuroscience.

[28] Seal RP (2009). Injury-induced mechanical hypersensitivity requires C-low threshold mechanoreceptors. Nature.

[29] Alvarez FJ, Villalba RM, Zerda R, Schneider SP (2004). Vesicular glutamate transporters in the spinal cord, with special reference to sensory primary afferent synapses. J Comp Neurol.

[30] Llewellyn-Smith IJ (2007). VGLUT1 and VGLUT2 innervation in autonomic regions of intact and transected rat spinal cord. J Comp Neurol.

[31] Elgarhy AK (2018). Immunohistochemical Expression of Synaptophysin in the Adult and Developing Cervical and Lumbar Enlargements of the Spinal Cord of Rabbit. Acad. Anat. Int..

[32] Wilson P, Kitchener PD (1996). Plasticity of cutaneous primary afferent projections to the spinal dorsal horn. Progress in neurobiology.

[33] Woodbury CJ, Kullmann FA, McIlwrath SL, Koerber HR (2008). Identity of myelinated cutaneous sensory neurons projecting to nocireceptive laminae following nerve injury in adult mice. J Comp Neurol.

[34] Ribeiro-da-Silva A, Coimbra A (1982). Two types of synaptic glomeruli and their distribution in laminae I-III of the rat spinal cord. J Comp Neurol.

[35] Ribeiro-da-Silva A, Tagari P, Cuello AC (1989). Morphological characterization of substance P-like immunoreactive glomeruli in the superficial dorsal horn of the rat spinal cord and trigeminal subnucleus caudalis: a quantitative study. J Comp Neurol.

[36] Todd AJ (2010). Neuronal circuitry for pain processing in the dorsal horn. Nat Rev Neurosci.

[37] Goldstein IJ, Blake DA, Ebisu S, Williams TJ, Murphy LA (1981). Carbohydrate binding studies on the Bandeiraea simplicifolia I isolectins. Lectins which are mono-, di-, tri-, and tetravalent for N-acetyl-D-galactosamine. J Biol Chem.

[38] Laitinen L (1987). Griffonia simplicifolia lectins bind specifically to endothelial cells and some epithelial cells in mouse tissues. Histochem J.

[39] Streit WJ, Kreutzberg GW (1987). Lectin binding by resting and reactive microglia. Journal of neurocytology.

[40] Chen J (2010). The extracellular matrix glycoprotein tenascin-C is beneficial for spinal cord regeneration. Mol Ther.

[41] Lee HJ (2012). Delayed applications of L1 and chondroitinase ABC promote recovery after spinal cord injury. J Neurotrauma.

[42] Lee HJ, Wu J, Chung J, Wrathall JR (2013). SOX2 expression is upregulated in adult spinal cord after contusion injury in both oligodendrocyte lineage and ependymal cells. J Neurosci Res.

